# Zika virus infection elicits auto-antibodies to C1q

**DOI:** 10.1038/s41598-018-20185-8

**Published:** 2018-01-30

**Authors:** Takaaki Koma, Veljko Veljkovic, Danielle E. Anderson, Lin-Fa Wang, Shannan L. Rossi, Chao Shan, Pei-Yong Shi, David W. Beasley, Natalya Bukreyeva, Jeanon N. Smith, Steven Hallam, Cheng Huang, Veronika von Messling, Slobodan Paessler

**Affiliations:** 1 Department of Pathology, University of Texas Medical Branch, Galveston, Texas USA; 2 Institute for Human Infections and Immunity, University of Texas Medical Branch, Galveston, Texas USA; 3 Biomed Protection, Galveston, Texas USA; 4 Programme in Emerging Infectious Diseases, Duke-NUS Medical School, Singapore, Singapore; 5 Department of Biochemistry & Molecular Biology, University of Texas Medical Branch, Galveston, Texas USA; 6 Institute for Translational Science, University of Texas Medical Branch, Galveston, Texas USA; 7 Sealy Center for Structural Biology & Molecular Biophysics, University of Texas Medical Branch, Galveston, Texas USA; 8 Department of Pharmacology & Toxicology, University of Texas Medical Branch, Galveston, Texas USA; 9 Department of Microbiology and Immunology, University of Texas Medical Branch, Galveston, Texas USA; 10 Veterinary Medicine Division, Paul-Ehrlich-Institute, Langen, Germany; 11 Present Address: Department of Microbiology, Tokushima University Graduate School of Medical Science, Tokushima, Japan

## Abstract

Zika virus (ZIKV) causes mostly asymptomatic infection or mild febrile illness. However, with an increasing number of patients, various clinical features such as microcephaly, Guillain-Barré syndrome and thrombocytopenia have also been reported. To determine which host factors are related to pathogenesis, the E protein of ZIKV was analyzed with the Informational Spectrum Method, which identifies common information encoded by primary structures of the virus and the respective host protein. The data showed that the ZIKV E protein and the complement component C1q cross-spectra are characterized by a single dominant peak at the frequency F = 0.338, suggesting similar biological properties. Indeed, C1q-specific antibodies were detected in sera obtained from mice and monkeys infected with ZIKV. As C1q has been known to be involved not only in immunity, but also in synaptic organization and different autoimmune diseases, a ZIKV-induced anti-C1q antibody response may contribute to the neurological complications. These findings might also be exploited for the design of safe and efficacious vaccines in the future.

## Introduction

Zika virus (ZIKV), a member of the family *Flaviviridae*, is the causative agent of Zika fever. Most cases are asymptomatic or patients develop mild symptoms with a maculopapular rash^1^. However, due to the rapid increase of ZIKV infections causing fetal deaths and more than 4,000 microcephaly cases and neurological disorders in affected areas, the World Health Organization (WHO) declared a public health emergency of international concern on February 1, 2016^2^. Although ZIKV was discovered nearly 70 years ago, data on the interaction of this virus with host targets is lacking and viral pathogenesis is still poorly understood. Currently, there is no prophylactic vaccine or effective therapy for Zika fever available.

To determine if there are host proteins with a pattern similar to the ZIKV envelope (E) glycoprotein, we analyzed this protein with informational spectrum method (ISM). Our analysis identified C1q, a member of the complement complex, as a host protein with common informational features, suggesting some similarity in biological properties. In addition to its role as the initial component of the classical complement cascade, C1q is known to be not only involved in innate immunity but also in homeostasis, angiogenesis, apoptosis, autoimmunity and synaptic organization^3–5^. Here, we demonstrate that ZIKV infection elicited antibodies specific to C1q in mice and non-human primates (NHP), which may contribute to the observed neurological complications.

## Results

### Human C1q shares spectral features with ZIKV but not West Nile virus E protein

**Figure 1:**
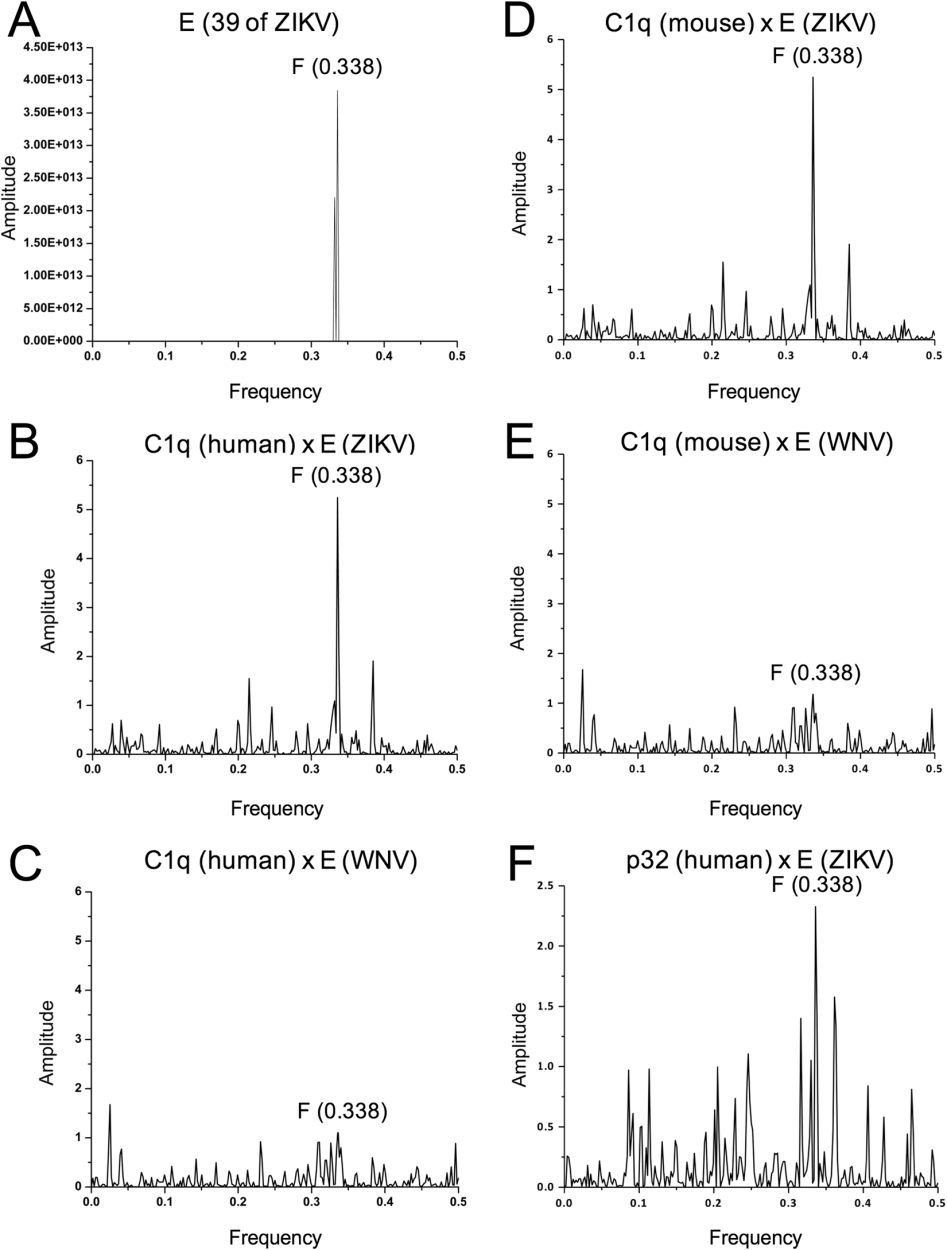
The ISM analysis of ZIKV E protein and C1q protein. (**A**) The consensus informational spectrum of 39 ZIKV E proteins (Supplementary dataset 1). (**B**) The cross-spectrum of E protein from ZIKV [strain H/PF/2013 (KJ776791)] and human C1q protein (NP_758957). (**C**) The cross-spectrum of WNV E protein (ACV44196) and the human C1q protein. (**D**) The cross-spectrum of E protein from ZIKV and murine C1q protein (NP_031600). (**E**) The cross-spectrum of WNV E protein and the murine C1q protein. (**F**) The cross-spectrum of E protein from ZIKV and human p32 protein (Q07021). The abscissa represents ISM frequencies, the ordinates are normalized amplitudes corresponding to each frequency component.

The consensus informational spectrum of 39 ZIKV E proteins (Fig. 1A and Supplementary dataset 1) was characterized, identifying a conserved dominant peak at the frequency F = 0.338. A dominant peak at this frequency was also found in the cross-spectrum between ZIKV E protein and human C1q (Fig. 1B). In contrast, the E protein of West Nile virus (WNV), another flavivirus, did not share spectral features with human C1q in the ISM analysis (Fig. 1C). Similar results were observed with mouse C1q and E proteins of ZIKV and WNV (Fig. 1D,E). This indicates that ZIKV E protein may specifically modulate the function of the human complement system *via* induction of E protein-specific antibodies that also cross-react with C1q.

### ZIKV infection induces C1q-specific antibodies

To assess if the similar profiles observed in ISM analysis translate into antibody cross-reactivity, we quantified anti C1q-specific antibodies using ELISA system.

**Figure 2:**
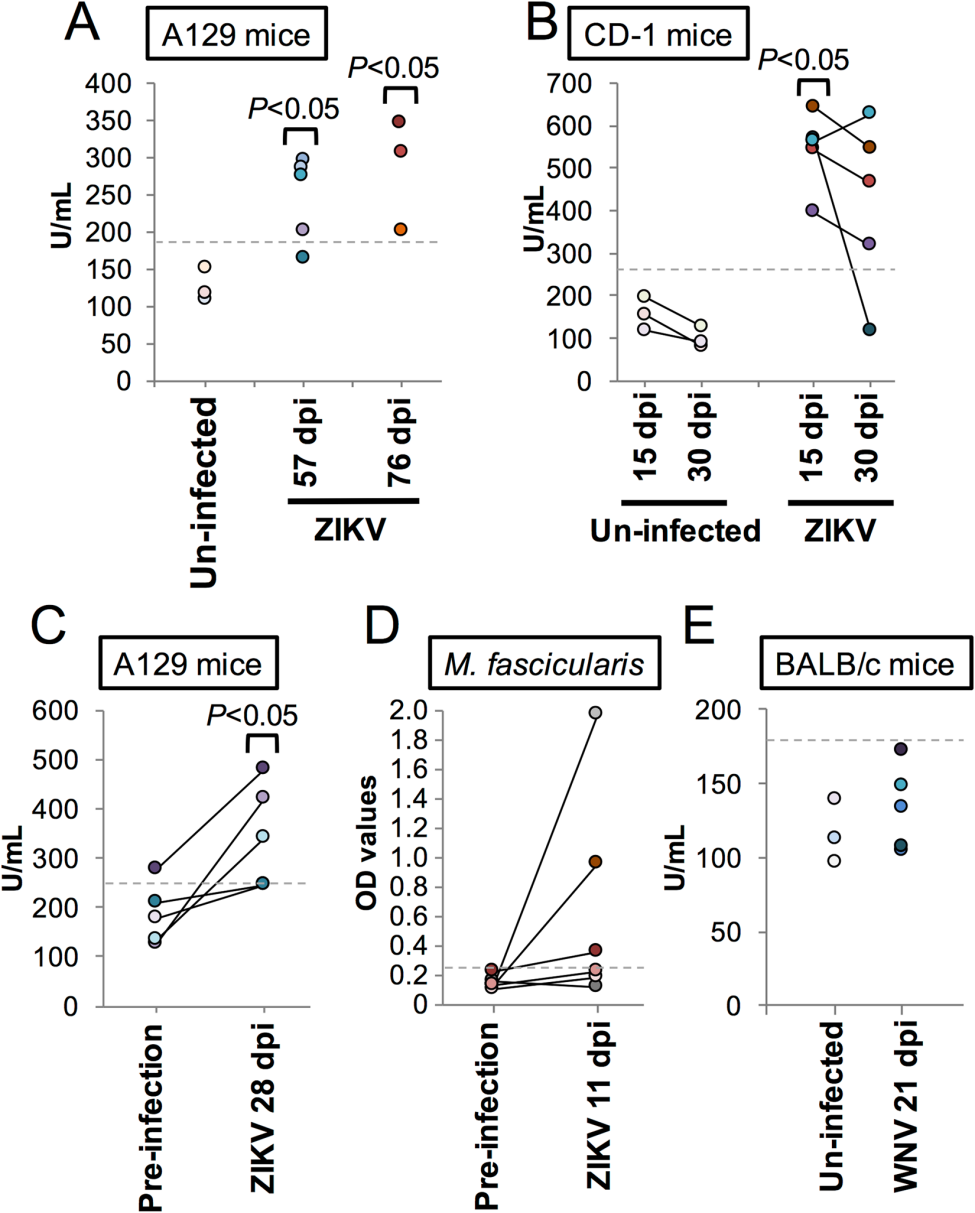
Anti-C1q antibody ELISA. (**A**) A129 mice were infected with ZIKV strain FSS13025 and sacrificed at indicated days. Anti-C1q antibody was measured by ELISA. The cut-off value was 190.0 U/mL (dash line). (**B**) Serum samples were collected from CD-1 mice infected with ZIKV strain FSS13025 and measured for anti-C1q antibody. The cut-off values were 268.4 U/mL (dash line) for 15 dpi and 205.8 U/mL for 30 dpi. (**C**) Serum samples were collected at 0 dpi (Pre-infection) and 28 dpi from A129 mice infected with ZIKV strain FSS13025. The dash lines indicate the cut-off value 252.1 U/mL. (**D**) Serum samples were collected at 0 dpi (Pre-infection) and 11 dpi from cynomolgus macaques (*Macaca fascicularis*) infected with ZIKV strain H/PF/2013. The dash lines indicate the cut-off value 0.272. The data is shown by ELISA OD values since there was no calibration samples in the ELISA kit. (**E**) Serum samples were collected from BALB/c mice infected with WNV TX AR12–9793 at 21 dpi and measured for anti-C1q antibody. The cut-off values were 178.2 U/mL (dash line). Each dot shows the average values of duplicate wells. The lines between dots indicate that the samples were obtained form same animals. P values were calculated using Mann-Whitney U test (compared with negative control or pre-infection). Each cut-off value was determined by the mean plus 3 SDs of the negative controls (un-infected) or pre-infection.

Immunodeficient A129 mice have been known to develop a disease after ZIKV inoculation in association with viremia, while immunocompetent CD-1 mice were not susceptible for ZIKV infection unless inoculated intracranially^6,7^. Therefore, to measure the immune responses we used both mouse models in our study. Serum samples were obtained from A129 mice lacking the type I interferon receptor as well as immunocompetent CD-1 mice on the indicated days after infection with the Cambodian ZIKV strain FSS13025. Seven out of 8 A129 mice displayed increased anti-C1q antibodies titers at 57 and 76 days post-infection (dpi) (Fig. 2A). Furthermore, a statistically significant increase in C1q-specific antibodies was observed in all five CD-1 mice at 15 dpi and the titer remained elevated in 4 animals until 30 dpi (Fig. 2B). Comparison of pre- and post-infection anti-C1q antibody levels in A129 mice revealed a significant increase at 28 dpi (Fig. 2C). To validate our findings in an animal model that is physiologically closer to humans, the presence of C1q-specific antibodies in serum samples from cynomolgus macaques infected with the Polynesian ZIKV strain H/PF/2013 strain was investigated using a macaque-specific ELISA kit. The analysis revealed an increase in C1q antibody levels in three out of the six animals at 11 dpi, when the animals were euthanized (Fig. 2D). Collectively, our results experimentally demonstrate that ZIKV infection can induce measurable levels of C1q cross-reactive antibodies not only in immunodeficient mice but also in immunocompetent mice and NHPs.

As ISM analysis demonstrated that the similarity with C1q is not shared with WNV E protein, we also quantified anti-C1q antibody response in WNV-infected immunocompetent BALB/c mice at 21 dpi. As predicted, even though all animals had neutralizing antibody titers against WNV of 1:320 or above, no increase in anti-C1q antibodies was detected (Fig. 2E).

### C1q shares spectral features with the E protein of another flavivirus, Yellow fever virus

**Figure 3:**
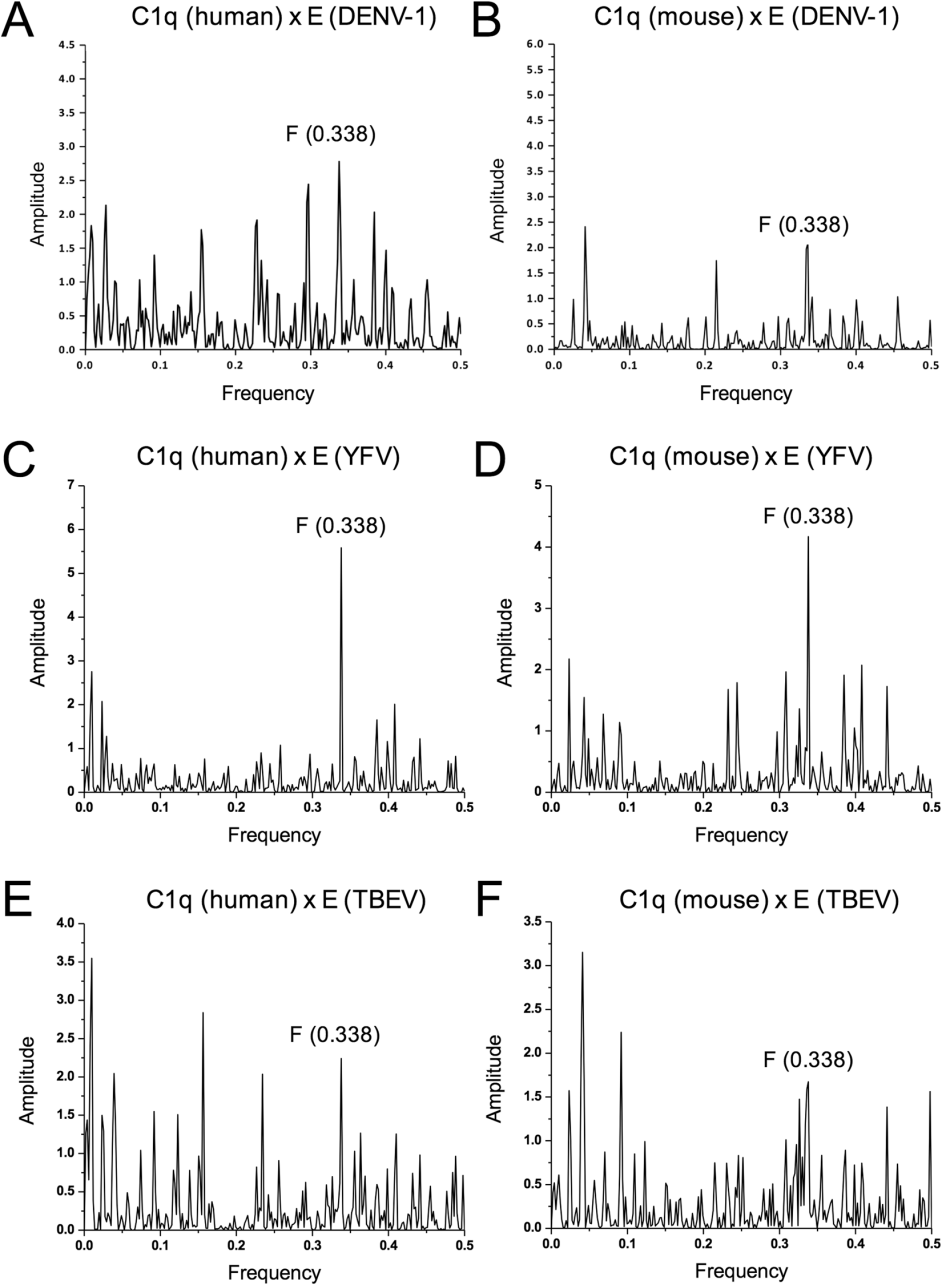
The ISM analysis of flavivirus E proteins and C1q protein. (**A**) The cross-spectrum of E protein from DENV-1 (P17763) and human C1q protein. (**B**) The cross-spectrum of E protein from DENV-1 and mouse C1q protein. (**C**) The cross-spectrum of E protein from YFV (AAX47570) and human C1q protein. (**D**) The cross-spectrum of E protein from YFV and mouse C1q protein. (**E**) The cross-spectrum of E protein from TBEV (AFV41132) and human C1q protein. (**F**) The cross-spectrum of E protein from TBEV and mouse C1q protein.

To explore if other flavivirus E proteins have similar properties to ZIKV, the E proteins of Dengue virus type 1 (DENV-1), Yellow fever virus (YFV) and tick-borne encephalitis virus (TBEV) were also analyzed by ISM method (Fig. 3). The DENV-1 E protein and C1q, and TBEV E protein and C1q are characterized by relatively low peak at the frequency F = 0.338. Compared to these data, the YFV E protein shares similar spectral features with both human and murine C1q like ZIKV E protein.

## Discussion

In this study, we demonstrated that animals infected with ZIKV develop antibodies against C1q, leading to speculation that these antibodies might contribute to ZIKV-associated complications such as microcephaly, thrombocytopenia and/or Guillain-Barré syndrome (GBS).

In the present study, we found informational similarities between C1q and the E protein of ZIKV using ISM, suggesting some biological similarity between these proteins. This similarity was not demonstrated for E protein from WNV. In accordance, we detected the elevated antibodies specific to C1q from almost all animals infected with ZIKV but not WNV.

Auto-antibodies to C1q have been frequently detected in patients with autoimmune diseases^8^, for examples, hypocomplementemic urticarial vasculitis (100%), Felty’s syndrome (76%), Lupus nephritis (63%) and systemic Lupus erythematosus (SLE) (33%). Conversely, anti-C1q antibodies can only be detected in 5–10% of “healthy” individuals and the etiology of their induction is unknown^8,9^.

The levels of anti-C1q antibodies often show a significant converse correlation with the level of C1q and C3^10–12^. Although the levels of C1q and C3 have not been measured in this study, the amount of C1q may decrease in the presence of high titers of anti-C1q antibodies titers. C1q and C3 are known to be involved not only in innate immunity but also brain development. Mice deficient in C1q or C3 have impaired synapse elimination during development, indicating that C1q and C3 are required for normal synaptic pruning^13^. Another study also reported that C1q is required for synapse elimination in the cortical layer^14^, and a case report showed that a girl was born with head circumference < 3rd percentile concomitantly with very low levels of C1q due to a mutation in the *C1qB* gene^15^. Therefore, viral infections that elicit cross-reactive anti-C1q antibodies may not only affect the course of the acute disease, but also impair normal brain development and function long after virus clearance. The proposed molecular mimicry between E protein of ZIKV origin and C1q that was identified using ISM might thus contribute to ZIKV-related microcephaly seen in infected babies or other neurological syndromes.

Recently, it was suggested that one additional important manifestation of ZIKV disease is immune-mediated severe thrombocytopenia^16^. Interestingly, ISM screening of platelets antigens suggested a possible mechanism of ZIKV associated thrombocytopenia. ISM results showed that the complement component 1 Q subcomponent-binding protein (p32) might be a host protein interacting with ZIKV E protein and/or a potential target for anti-E antibodies through cross reaction (Fig. 1F). Furthermore, recent papers reported ZIKV-related GBS^17^ and Sensory Polyneuropathy^18^. Some evidence indicates that the complement components are the cause of GBS^19,20^. Anti-C1q antibodies or ZIKV infection may activate the complement components and contribute to the development of GBS.

C1q not only plays a role in immunity but also in homeostasis and development^3–5^. The C1q-mimicking ZIKV E protein might induce autoimmune response against C1q in host. This potential immune response may have implications in the pathogenesis of ZIKV, including development of microcephaly, GBS and thrombocytopenia. More investigations are warranted to confirm these hypotheses. Additionally, it would be important to confirm our data in Zika patients and compare anti-C1q antibody levels between people that were infected and those that have not been infected with ZIKV. In the ISM analysis for other flavivirus E proteins, YFV E protein also shared similar spectral features with both human and murine C1q. Although in present study we did not test the anti-C1q antibody inducibility by YFV infection or vaccine *in vivo*, future studies are warranted to test the anti-C1q antibody induction after YFV infection. Some cases of neurological diseases such as encephalitis and GBS (suspect) have been reported after Yellow fever vaccination^21–23^. It would be interesting to study a potential role of YFV-elicited anti-C1q antibody in YFV-associated neurological diseases.

Additionally, as some ZIKV vaccines are entering clinical trials, we believe that it is important to ensure that these vaccines would not induce anti-C1q antibodies, which may mediate autoimmune response in human vaccinees. If the induction of anti-C1q antibodies is identified in vaccinees, safer vaccines should be designed to avoid a risk of eliciting autoimmune responses in healthy humans after vaccination.

## Methods

### Informational spectrum method (ISM)

ISM technique has been described in detail previously^24^. The ISM is a virtual spectroscopy-based analysis of protein-protein interactions. Amino acid sequences are transformed into signals by assigning numerical values of an amino acid element in the method^24^. These values reflect the electron-ion interaction potential, which determines the electronic properties of amino acids^25,26^. The obtained signal is then decomposed in periodical function by Fourier transformation. The result is displayed by a series of frequencies that correspond to the distribution of structural motifs with defined physico-chemical characteristics. Proteins with overlapping peaks or pattern thus share the biological or biochemical functions^24^.

### Viruses and animals

ZIKV Cambodian strain FSS13025 (10^5^PFU) was intraperitoneally inoculated into IFN-αβ R^−/−^ mice (A129 mice) which were bred and maintained in the University of Texas Medical Branch at Galveston and CD-1 mice which were purchased from Charles River Laboratories (Wilmington, MA). Intraperitoneal administration was previously used to establish ZIKV infection model in mice. All experiments with cynomolgus macaques (*Macaca fascicularis*) were conducted at SingHealth Experimental Medicine Centre. ZIKV Polynesian strain H/PF/2013 (10^5^PFU) was administrated into *M*. *fascicularis* by intravenous or subcutaneous routes that mimic mosquito bites. Although different ZIKV strains were used in mice and NHP experiments, they were genetically very similar (amino acid sequence similarity and identity: 99.9% and 99.6%, respectively) and phylogenetically classified into the recent epidemic Asian linage^27^. The experiments with mice and NHP were approved by the Institutional Animal Care and Use Committee at the University of Texas Medical Branch at Galveston and the Institutional Animal Care and Use Committee of the SingHealth Experimental Medicine Centre, respectively. All methods in this study were performed in accordance with relevant guidelines and regulations. Sera which were previously obtained from WNV-infected animals^28^ were used to detect anti-C1q antibodies.

### ELISA

To detect anti-C1q specific antibodies, “Mouse Anti C1q Ig’s ELISA kit” (Alpha Diagnostic International, San Antonio, TX) and “Human Anti-Complement 1q antibody ELISA kit” (MyBiosource, San Diego, CA) were used for mouse and NHP, respectively. ELISA was carried out according to the manufacturer’s protocols. For NHP, the human IgG HRP conjugated secondary antibody was substituted with a monkey IgG HRP conjugated secondary antibody.

## Electronic supplementary material

Dataset 1
